# Thymol as a Potential Natural Antiemetic: Insights From In Vivo and In Silico Studies

**DOI:** 10.1002/fsn3.70832

**Published:** 2025-09-10

**Authors:** Showkoth Akbor, Mehedi Hasan Bappi, Abdullah Al Shamsh Prottay, Farjanamul Haque, Nayem Mia, Tawhida Islam, Zainab M. Almarhoon, Eda Sönmez Gürer, William N. Setzer, Javad Sharifi‐Rad, Sakib Al Hasan, Muhammad Torequl Islam

**Affiliations:** ^1^ Department of Pharmacy Gopalganj Science and Technology University Gopalganj Bangladesh; ^2^ Department of Chemistry, College of Science King Saud University Riyadh Saudi Arabia; ^3^ Sivas Cumhuriyet University, Faculty of Pharmacy Department of Pharmacognosy Sivas Turkey; ^4^ Aromatic Plant Research Center Lehi Utah USA; ^5^ Department of Chemistry University of Alabama in Huntsville Huntsville Alabama USA; ^6^ Universidad Espíritu Santo Samborondón Ecuador; ^7^ Centro de Estudios Tecnológicos y Universitarios del Golfo Veracruz Mexico; ^8^ Department of Medicine, College of Medicine Korea University Seoul Republic of Korea; ^9^ Bioinformatics and Drug Innovation Laboratory BioLuster Research Center Ltd. Gopalganj Bangladesh; ^10^ Pharmacy Discipline Khulna University Khulna Bangladesh

**Keywords:** 5‐HT_3_ antagonist, calcium channel, GABA_B_ agonist, nausea and vomiting, thymol

## Abstract

Nausea and vomiting are common and distressing responses to toxins, chemotherapy, and gastrointestinal disturbances. Although antiemetic medications are available, their adverse effects make safer substitutes necessary. In our study, Thymol (THY), a phenolic monoterpene from essential oils, was evaluated for its antiemetic potential using in vivo and *in silico* methods. In the in vivo study, emesis was induced in 2‐day‐old chicks by oral administration of copper sulfate pentahydrate (50 mg/kg). THY was administered orally at doses of 10, 20, and 40 mg/kg, alone or in combination with standard antiemetics ondansetron (ODN), domperidone (DPD), hyoscine butyl bromide (HYS), and promethazine hydrochloride (PRO). The 20 mg/kg dose (THY‐20) showed the highest efficacy, significantly (*p* < 0.0001) reducing the number of retches (24.6 ± 2.7; 67.2% reduction) and increasing latency to first retch (52.6 ± 4.2 s; 77.18% increase) compared to the negative control (NC). The THY + ODN combination further enhanced effects (68.53% retch reduction). Molecular docking showed strong binding of THY to 5‐HT_3A_ (−6.4 kcal/mol), D_2_ (−7.1 kcal/mol), M_3_ (−6.2 kcal/mol), and H_1_ (−7.1 kcal/mol) receptors, comparable to standard drugs. Absorption, distribution, metabolism, excretion, and toxicity (ADMET) profiling revealed THY's compliance with Lipinski's Rule, high gastrointestinal absorption, blood–brain barrier permeability, and low toxicity risk. The multi‐target binding profile and synergistic potential of THY suggest its promise as a natural, broad‐spectrum antiemetic. Further receptor‐specific studies and trials in chemotherapy‐induced emesis models are recommended to validate its clinical potential.

## Graphical abstract

According to the study, thymol (THY) greatly decreased retches and enhanced latency in a model of chick emesis; the highest effectiveness was shown at 20 mg/kg. According to molecular docking, THY binds strongly to important emetic receptors (5‐HT_3A_, D_2_, M_3_, and H_1_), indicating a multi‐target mechanism. THY's favorable ADMET characteristics and synergistic effects with conventional medications make it a viable natural antiemetic option.

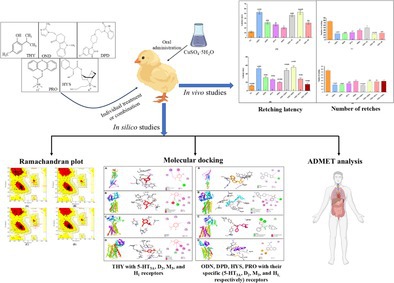

## 1 Introduction

Nausea and vomiting are crucial protective defenses generally occurring by uncomfortable action that causes the evacuation of stomach contents via the mouth or, occasionally, the nose and are unmistakably related to gastrointestinal motor activity (Zhong et al. 2021). It is a biological system's response to adverse drug effects, excessive motion stimuli, visceral‐originated functional stimuli, chemical stimuli, illness co‐morbidities, and defense against food poisoning (Ahmed et al. 2014; Eghbali et al. 2016). The causes of vomiting and nausea can be toxic or infectious (bacteria, bacterial toxins, viruses, food‐borne toxins), gastrointestinal (functional disorder, peptic ulcer disease, malignancy, organic disorders, irritable bowel syndrome (IBS), chronic intestinal pseudo‐obstruction), central nervous system (motion sickness, migraine, cerebrovascular accident, seizure disorder), or psychiatric, like anorexia nervosa (Scorza et al. 2007; Kuo and Singh 2016; Fejzo et al. 2019). The adverse impacts of chemotherapy and radiation therapy treatments usually include nausea and vomiting (Mahesh et al. 2005). By activating the vomiting center (VC), motor pathways descend from this center and trigger vomiting. Pro‐emetic impulses to the VC can come from the abdominal vagal and glossopharyngeal afferents, the vestibular system, the central nervous system, and chemical emotegens in the circulation via the area postrema, also referred to as the chemoreceptor trigger zone (CTZ) (Sanger and Andrews 2006). As a central generator, the VC activates brainstem output nuclei in a controlled manner to trigger the emetic reaction in response to inputs that exceed the emetic threshold (Hornby 2001).

**FIGURE 1:**
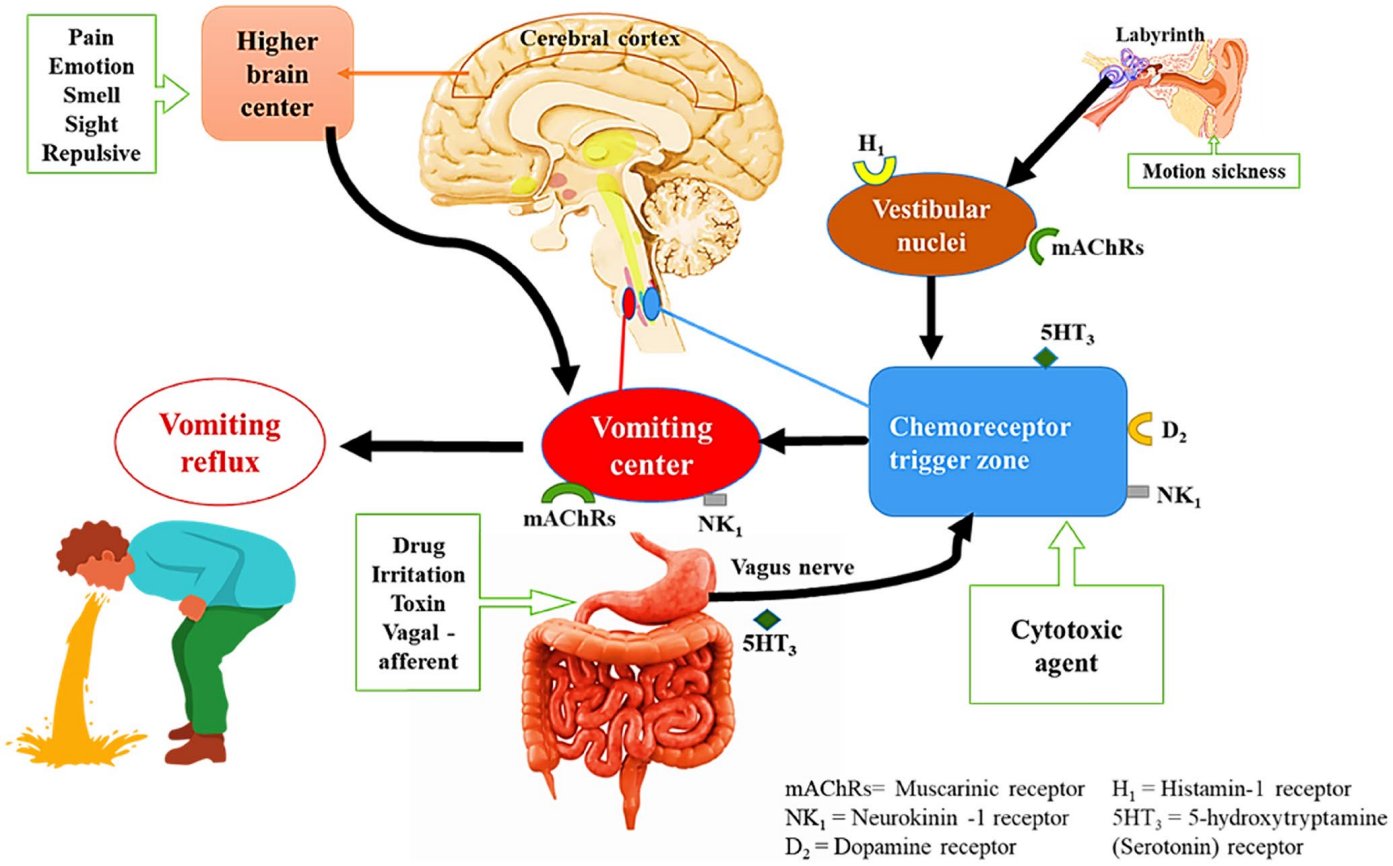
Mechanisms that can trigger nausea and vomiting.

The gastrointestinal tract, thalamus, cerebral cortex, vestibular region, and CTZ are the four main sites that can either directly or indirectly activate the VC (Becker 2010). Vomiting factors can act directly on the locations that they target or indirectly by releasing agents or emetic neurotransmitters that stimulate the appropriate emetic receptors, such as neurokinin 1, serotonin type 3 (5‐HT_3_), dopamine D_2_ and D_3_, opioid mu, and kappa, muscarinic (M_1_), and histamine (H_1_). The histaminergic neural system activated during motion sickness triggers the brainstem's histamine H_1_ receptors to cause vomiting (Chen et al. 2018). The limbic system and cerebral cortex both receive emotional and cognitive stimuli that make individuals desire to vomit (Cai et al. 2007; Cohen et al. 2019). Several processes might cause nausea and vomiting, as depicted in Figure 1.

Serotonin receptor antagonists (selective 5‐HT_3_ receptor antagonists) like ODN, granisetron, etc. are generally well tolerated, but constipation, headache, dizziness, and temporary increases in serum transaminase are some of their adverse effects (Hendren et al. 2015). Dopamine receptor antagonists (DPD) can cause drowsiness, anxiety, mood swings, and insomnia, which can cause breast enlargement and sexual dysfunction (Schey et al. 2016). The use of antihistamines such as diphenhydramine, hydroxyzine, cinnarizine, and others may be restricted in specific situations due to sedation and dry mouth. Muscarinic receptor antagonists, such as scopolamine, can cause constipation, dry mouth and eyes, headaches, drowsiness, poor concentration, and urine retention as adverse effects (Hendren et al. 2015). An NK1 receptor antagonist may cause manic mental illness, anxiety, sadness, and visual hallucinations (Navari 2018).

Thymol (THY) is a natural phenolic monoterpene and volatile oil component (Jyoti et al. 2019). Researchers have recently discovered monoterpenes to have antiemetic effects (Boğa et al. 2019). THY exhibits antimicrobial, anti‐carcinogenic (Deb et al. 2011), anti‐inflammatory (Fachini‐Queiroz et al. 2012), immune modulator (Salehi et al. 2018), antiseptic, antibacterial (Ghizlane et al. 2011), antifungal (Shen et al. 2016), antiviral (Kazemi Oskuee et al. 2011), antioxidant (Al‐Malki 2010), immunomodulatory (Hashemipour et al. 2013), analgesic (Ghori et al. 2016), sedative, anti‐rheumatic, anti‐cancer, and anti‐hyperlipidemic properties (Kensara et al. 2013; Yu et al. 2016). THY is corrosive to the skin and eye (Toxicity Category I), and ingestion may cause burning pain at high doses. In the acute toxicity test in mice, the LD_50_ of THY was 1350.9 mg/kg (André et al. 2017). The aqueous, methanolic, and petroleum ether extracts of the aerial parts of *Thymus transcaspicus Klokov* exhibited potent antiemetic activity, and THY is its major chemical constituent (Moallem et al. 2009). Some studies suggest that THY acts as a 5‐HT_3_ antagonist (Bhandari and Kabra 2014; Parker et al. 2014). However, there is not yet an established antiemetic activity of THY. The first time we experimented on THY in a chick model was to find out whether this compound had any antiemetic effect. This study aims to evaluate the antiemetic effect of THY on copper sulfate pentahydrate‐induced emesis in chicks and *in silico* studies to find out possible involved receptors.

## 2 Materials and Methods

### 2.1 In Vivo Study

#### 2.1.1 Chemicals and Reagents

**FIGURE 2:**
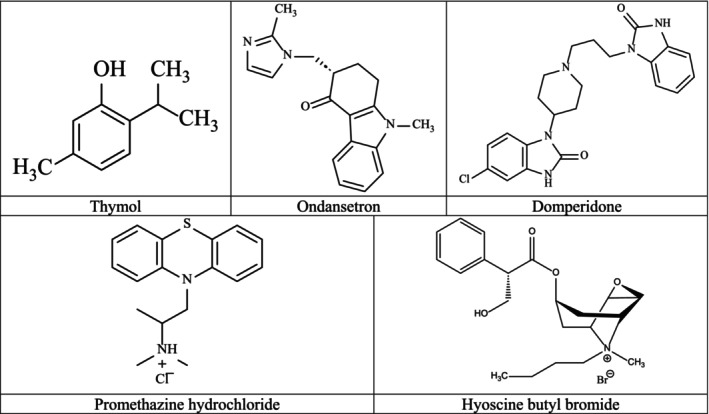
The two‐dimensional chemical structures of test sample and standard drugs.

THY (5‐methyl‐2‐(propane‐2‐yl) phenol, purity ≥ 98.5%, T0501‐100G, Lot #SLCB5100, Pcode: 1002887049 (product of India), CAS No. 89–83‐8) was purchased from Sigma‐Aldrich (USA), and 0.05% tween 80 (CAS No. 9005‐65‐6) and copper sulfate pentahydrate (CuSO_4_.5H_2_O) (CAS No. 7758‐99‐8) were bought from Merck (India). DPD and ODN as reference drugs were purchased from Team Pharma Ltd. and Incepta Pharma Ltd., respectively. HYS and PRO were both obtained from Opsonin Pharma Ltd., Bangladesh. The two‐dimensional chemical structures of the test and the standard drugs are shown in Figure 2.

#### 2.1.2 Experimental Animals

For this study, 2‐day‐old, male or female *Galactosaurus domesticus* chicks (Grade‐A) weighing approximately 40–42 g were obtained from Nourish Grand Parent Ltd. in Rangpur, Bangladesh. The chicks were housed in the pharmacology lab of Gopalganj Science and Technology University in Gopalganj. Regular food and water were provided to the animals without restriction. Up to the start of the test, they were maintained at 27°C ± 1°C with regulated illumination (12 h of light and dark cycles). The experiment was conducted daily from 8:00 a.m. to 3:00 p.m., and the animals were monitored for 70 h post‐treatment to observe any signs of toxicity or mortality. Importantly, the decision to use a small sample size (*n* = 5 per group) was based on ethical and scientific considerations. In alignment with the internationally recognized 3Rs principle, Replacement, Reduction, and Refinement, we aimed to minimize animal usage without compromising the reliability of our results. The number of animals used was deemed sufficient to detect preliminary pharmacological effects, especially in light of similar precedents in published studies. This approach ensures both humane treatment of animals and adherence to scientifically justified protocols. This study was approved by the Animal Ethics Committee of Khulna University (KUAEC‐2023‐05‐09).

#### 2.1.3 Study Design

With a few slight modifications, the in vivo investigation was conducted by Akita et al.'s (1998) guidelines (Akita et al. 1998). The test was run at 27°C ± 1°C temperature, and all animals were healthy. Twelve groups, each containing five chicks, were created from all of the individuals (Table 1).

**TABLE 1:** Description of treatment groups, dose, and route of administration.

Treatment groups	Description	Test dose
NC	Vehicle (0.05% Tween 80 + 0.9% NaCl solution)	10 mL/kg
ODN	Standard 1: Ondansetron [Serotonin (5‐HT_3A_) antagonist]	24 mg/kg
DPD	Standard 2: Domperidone [Dopamine‐2 (D_2_) antagonist]	80 mg/kg
HYS	Standard 3: Hyoscine Butyl‐bromide [Muscarinic‐3 (M_3_) antagonist]	100 mg/kg
PRO	Standard 4: Promethazine Hydrochloride [Histamine‐1 (H_1_) antagonist]	100 mg/kg
THY‐10	Test sample: Thymol‐10 (Lower dose)	10 mg/kg
THY‐20	Test sample: Thymol‐20 (Middle dose)	20 mg/kg
THY‐40	Test sample: Thymol‐40 (Upper dose)	40 mg/kg
THY + ODN	Thymol+ Ondansetron	20 + 24 mg/kg
THY + DPD	Thymol + Domperidone	20 + 80 mg/kg
THY + HYS	Thymol + Hyoscine butyl bromide	20 + 100 mg/kg
THY + PRO	Thymol + Promethazine	20 + 100 mg/kg
*Vomiting inducer agents	Copper sulfate pentahydrate (CuSO_4_.5H_2_O)	50 mg/kg

Each chick was placed in a big, clear plastic container for 10 min before receiving the treatments. The doses of THY (10, 20, and 40 mg/kg) were carefully chosen to cover a low, moderate, and relatively higher range based on prior pharmacological research and to allow assessment of a potential dose‐dependent antiemetic response. This range was selected to explore both efficacy thresholds and possible saturation points for THY's biological activity in vivo (Saravanan and Pari 2015; Baldissera et al. 2018) and administered orally after being dissolved in a 0.9% sodium chloride solution containing a trace of 0.05% Tween 80. ODN, DPD, HYS, and PRO were administered orally as positive controls at doses of 24, 80, 100, and 100 mg/kg b.w., respectively (Bappi, Prottay, Al‐Khafaji, et al. 2023; Bappi, Prottay, Kamli, et al. 2023). Animals were also given four oral combinations of 20 mg/kg of THY‐20 and the positive controls ODN, DPD, HYS, and PRO at doses of 24, 80, 100 mg/kg, and 100 mg/kg b.w., respectively. The vehicle group was regarded as a negative control (NC). After 30 min of treatment, emesis was induced through CuSO_4_.5H_2_O at a dose of 50 mg/kg b.w. (Bappi, Prottay, Al‐Khafaji, et al. 2023; Bappi, Prottay, Kamli, et al. 2023) by administering it orally to every chick. Then the latency (first retching after having CuSO_4_.5H_2_O treatment) and the number of retches (within 20 min after having CuSO_4_.5H_2_O treatment) were carefully recorded. The percentage increase in latency and decrease in retches inspection of NC were calculated according to the following equations:

$$\%\text{Increase in latency}=\frac{A-B}{A}\times 100$$

$$\%\text{Decrease in retches}=\frac{C-D}{C}\times 100$$

where, *A* = Mean of latency in seconds in the NC group, *B* = Mean of latency in seconds in standard and test groups, *C* = Mean of retches in the NC group, and *D* = Mean of retches in standard and test groups.

#### 2.1.4 Statistical Analysis

Antiemetic activity values are presented as the mean ± standard deviation (SD). Data were tested for normality using the Shapiro–Wilk test. All datasets met the assumption of normal distribution (*p* > 0.05) and were analyzed using one‐way analysis of variance (ANOVA) followed by Tukey's post hoc test using the statistical software GraphPad Prism (version 9.5.0) and experimental groups are compared with the vehicle (control) group. Statistical significance was set at *p* < 0.05.

### 2.2 In Silico Study

#### 2.2.1 Macromolecules and Homology Model

**FIGURE 3:**
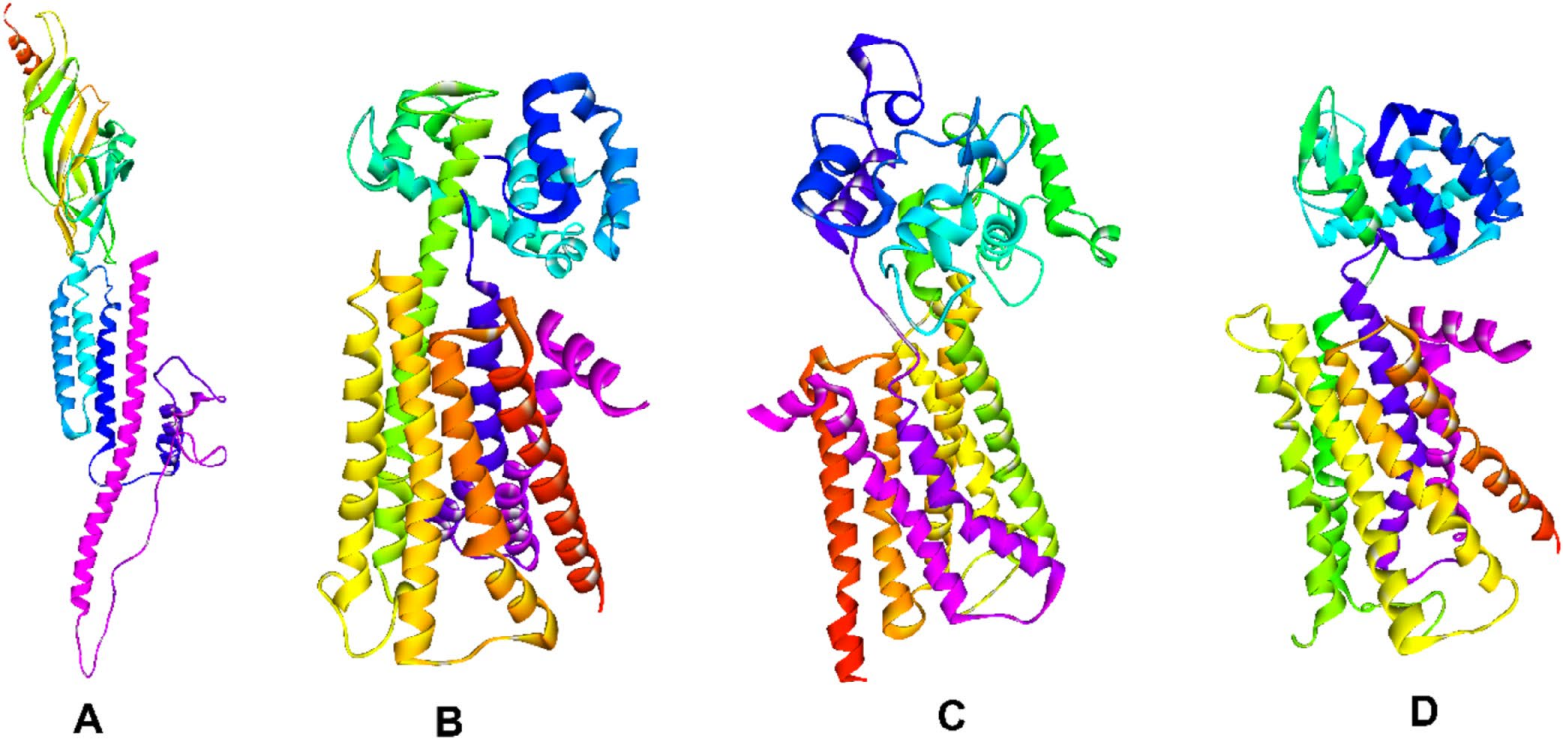
The three‐dimensional crystal structure of emetic receptors (A) 5‐HT_3A_, (B) D_2_, (C) M_3_, and (D) H_1_.

Homology modeling of the 5‐HT_3A_ (UniProt ID: P46098) and muscarinic M_3_ (P20309) receptors of *Homo sapiens* was carried out using the SWISS‐MODEL server (Kiefer et al. 2009), with optimal templates (SMTL ID: 4pir.1.A for 5‐HT_3A_ and 4daj.1.A for M_3_) selected via BLASTp against the Protein Data Bank (PDB). The crystal structures of dopamine D_2_ (PDB ID: 6CM4) and histamine H_1_ (3RZE) receptors were directly retrieved from the PDB (Rose et al. 2017). To assess model reliability, stereochemical validation was performed using PROCHECK‐generated Ramachandran plots, which showed that over 91% of residues in all models were in the most favorable regions (Gopalakrishnan et al. 2007). All structures were energy minimized using Swiss‐PDB Viewer (v4.1.0) prior to docking. Homology modeling, an essential tool in structural biology, facilitates accurate structure prediction by reducing gaps between known protein sequences and experimental data (Akdel et al. 2022; Martí‐Renom et al. 2000). Amino acid sequences were sourced from UniProt, and validation results supported the structural integrity of the models (Mia et al. 2023; Bappi et al. 2024). These validated structures were then used for molecular docking of THY and standard drugs to explore receptor binding mechanisms. Figure 3 shows the 3D structure of the emetic receptors. Before docking, the energy of emetic homology models was minimized using the Swiss‐PDB Viewer software (v 4.1.0), and the PROCHECK Ramachandran plot (Figure 3) was used to confirm their validity (Bappi et al. 2024).

#### 2.2.2 Ligand Preparation

The PubChem database was queried for the 3‐dimensional molecular structure for THY (PubChem ID: 6989), in addition to standard drugs ODN, DPD, HYS, and PRO (PubChem ID: 4595, 3151, 6852391, and 6014, respectively). Chem3D Pro20.1.1 software systems were used to optimize the internal energy of all the compounds (Akbor et al. 2023).

#### 2.2.3 Docking Protocol

Molecular docking was carried out using AutoDock Vina via PyRx (v0.8), a widely used virtual screening platform (Kamli et al. 2023). Docking parameters were standardized with a grid box size of 85 × 80 × 75 Å along the x, y, and z axes and an exhaustiveness value of 2000. The ligand‐protein complex is retrieved in PDB format for ligand collection in PDBQT format, and the docking potential result is recorded in “CSV” format. Active binding areas of proteins are found with PyMol v 1.7.4.5 (Bhuia et al. 2023) as the ligand values in the initial target protein grid systems, and these are investigated with BIOVIA Discovery Studio (version 21.1.0) (Bappi, Prottay, Al‐Khafaji, et al. 2023; Bappi, Prottay, Kamli, et al. 2023).

#### 2.2.4 ADMET Prediction

The SwissADME online platform (http://www.swissadme.ch/) was utilized for analyzing the physicochemical properties of effective candidates, including aqueous solubility, lipid affinity, and pharmacokinetics. ADMET properties of THY were predicted using the SwissADME online tool (Haque et al. 2024).

## 3 Results

### 3.1 In Vivo Study

**FIGURE 4:**
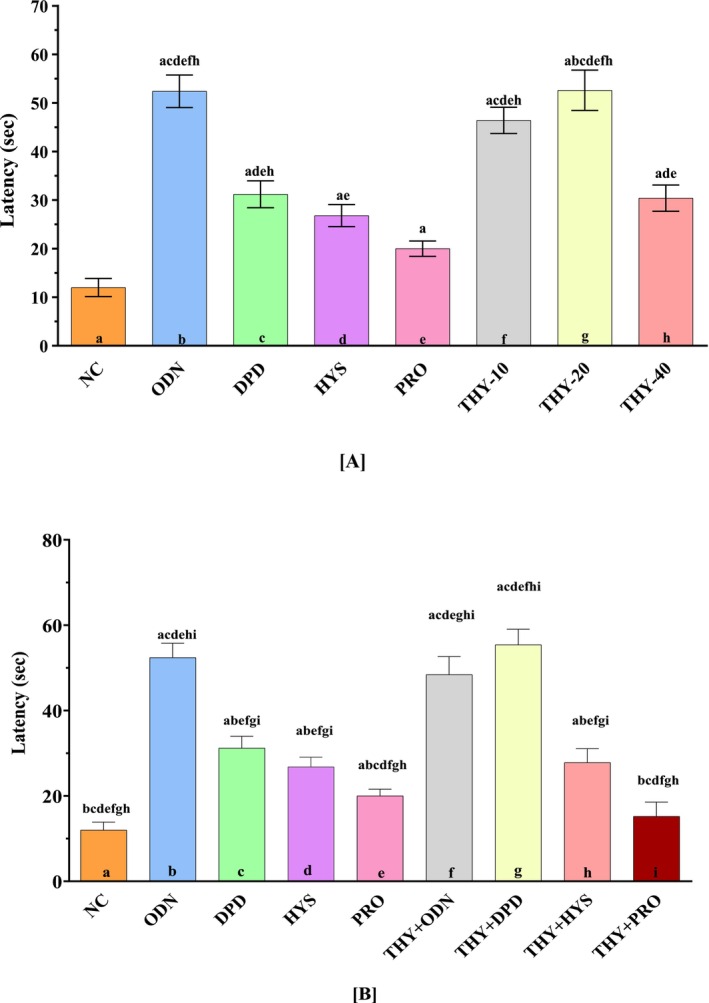
(A) Retching latency was noticed in single‐dose test samples and controls. (B) Retching latency was noticed in controls and combinations. Values are presented as mean ± SD (*n* = 5); data normality was confirmed using the Shapiro–Wilk test (*p* > 0.05). Statistical analysis was performed using one‐way ANOVA followed by Tukey's post hoc test for multiple comparisons. *p* < 0.05 compared to the ^a^NC (Vehicle); ^b^ODN; ^c^DPD; ^d^HYS; ^e^PRO; ^f^THY + ODN; ^g^THY + DPD; ^h^THY + HYS; ^i^THY + PRO; NC: Vehicle (distilled water containing 0.9% NaCl and 0.5% tween 80); DPD, domperidone; HYS, hyoscine butyl bromide; ODN, ondansetron; PRO, promethazine hydrochloride.

THY dosages significantly increased the latent period and lowered the number of retches in chicks. Prior to analysis, data normality was confirmed by the Shapiro–Wilk test (*p* > 0.05). THY‐20 increased significantly (*p* < 0.0001) latency compared to NC, DPD, HYS, and PRO (Figure 4). Retching started in the test groups at 46.4, 52.6, and 30.4 s for THY‐10, THY‐20, and THY‐40, in that order. The combination of THY and ODN increased significantly (*p* < 0.0001) latency compared to NC, THY, DPD, ODN, PRO, HYS, and THY + HYS. In the THY + DPD group, the initial retching was seen at 55.4 s (mean value) (Figure 4).

**FIGURE 5:**
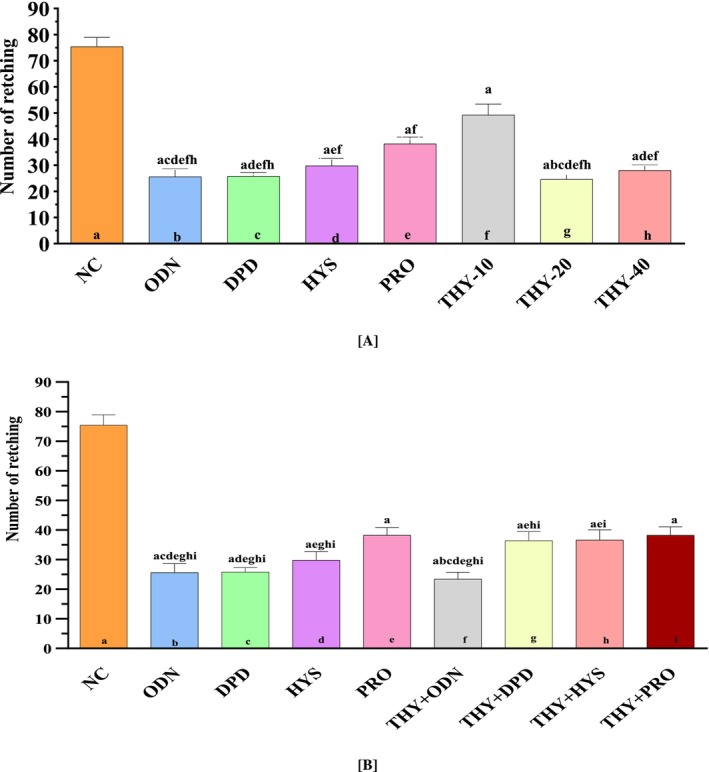
(A) Number of retching noticed in single doses test samples, and controls. (B) Retching latency was noticed in controls, and combinations. Values are presented as mean ± SD (*n* = 5); data normality was confirmed using the Shapiro–Wilk test (*p* > 0.05). Statistical analysis was performed using one‐way ANOVA followed by Tukey's post hoc test for multiple comparisons. *p* < 0.05 compared to the ^a^NC (Vehicle); ^b^ODN; ^c^DPD; ^d^HYS; ^e^PRO; ^f^THY + ODN; ^g^THY + DPD; ^h^THY + HYS; ^i^THY + PRO; NC: Vehicle (distilled water containing 0.9% NaCl and 0.5% tween 80); DPD, domperidone; HYS, hyoscine butyl bromide; ODN, ondansetron; PRO, promethazine hydrochloride.

The NC group revealed the highest retching (mean value: 75.4). The test groups THY reduced the number of retches; the values were 49.2, 24.6, and 31 for the THY10, THY‐20, and THY‐40 groups, respectively, which were comparatively lower than the NC group. THY‐20 decreased a significant (*p* < 0.0001) number of retching's. The combined groups reduced a significant (*p* < 0.0001) number of retches compared to NC, HYS, PRO, THY + DPD, THY + HYS, and THY + PRO. Within the THY + ODN group, the least number of retches was seen (Figure 5).

The percentage increase in latency inspection of NC for the THY‐10, THY‐20, and THY‐40 groups was observed at 74.13%, 77.18%, and 60.52%, respectively. On the other hand, the percentage reduction in test group retches compared to NC was 34.3%, 67.2%, and 58.6% for the THY‐10, THY‐20, and THY‐40 groups, respectively. The maximum percentage enhancement in latency for the THY‐10 was 77.18%, and the increased latency was 67.2% for the THY‐20 group with respect to the NC group. In the combined groups compared to NC, the maximum percentage reduction in retches (68.53%) was seen in the THY + ODN group, and the peak percentage increase in latency (78.33%) was seen in the THY + DPD group (Table 2).

**TABLE 2:** Modulatory latency and retches by the test and/or controls.

Treatment groups	%Increase in latency	%Decrease in retches
NC	—	—
ODN	77.09	65.86
DPD	61.53	64.80
HYS	55.22	60.26
PRO	40.00	49.06
THY‐10	74.13	34.40
THY‐20	77.18	67.20
THY‐40	60.52	58.66
THY + ODN	75.20	68.53
THY + DPD	78.33	51.46
THY + HYS	56.83	50.66
THY + PRO	21.05	49.06

*Note:* Values are percentage inspect of control group (Negative control or vehicle) (*n* = 5); ODN = ondansetron (24 mg/kg); DPD = domperidone (80 mg/kg); HYS = hyoscine (100 mg/kg); PRO = promethazine (100 mg/kg); THY‐10 = thymol (10 mg/kg); THY‐20 = thymol (20 mg/kg); THY‐40 = thymol (40 mg/kg); THY + ODN = thymol and ondansetron (20 + 24 mg/kg); THY + DPD = thymol and domperidone (20 + 24 mg/kg); THY + HYS = thymol and hyoscine (20 + 100 mg/kg); THY + PRO = thymol and promethazine (20 + 100 mg/kg).

### 3.2 In Silico Study

#### 3.2.1 Ramachandran Plot

**FIGURE 6:**
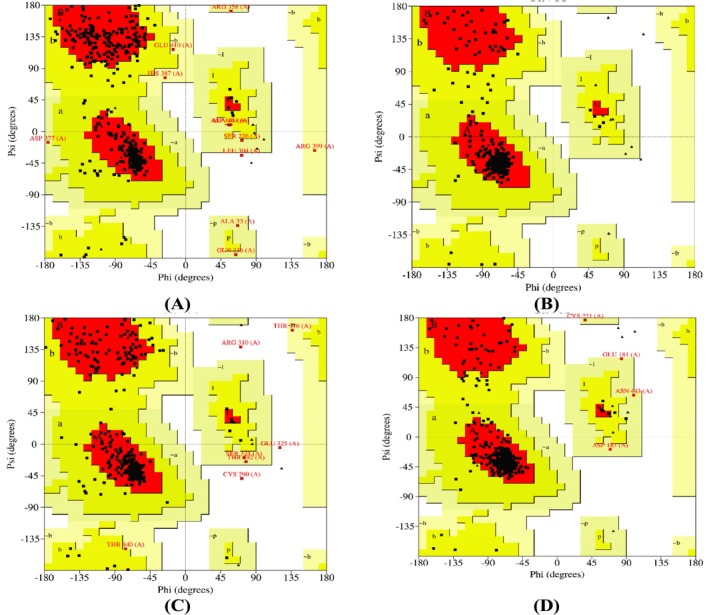
Ramachandran plot of (A) 5‐HT_3A_, (B) D_2_, (C) M_3_, and (D) H_1_.

The Ramachandran plot is a fast process to see the pattern of torsion angles in such a protein structure. Furthermore, it indicates the acceptable and prohibited torsion angle values for assessing the caliber of three‐dimensional protein structures (Prottay et al. 2024). The Ramachandran plot depicts the Φ‐Ψ torsion angles for each residue (except those at the chain termini). The many components that have been discussed are represented by the color and shade of the map; the “core” or dark regions (marked in red) correspond to the most beneficial pairings of Π and Ψ ratios. Ideally, more than 90% of the residues would be included in these “core” pieces. The residues in the most favorable areas for 5‐HT_3A_, D_2_, M_3_, and H_1_ range approximately 91.65%, 92.60%, 92.67%, and 92.10%, according to Ramachandran plot estimates. The residue percentages in “core” locations are one of the most reliable measures of stereochemical integrity (Figure 6).

**TABLE 3:** Binding affinity values and interaction residues of thymol with vomiting receptors.

Thymol (THY) & protein (receptor)	Binding affinity (kcal/mol)	H‐bond		Hydrophobic bond	
		Residues	Bond types	Residues	Bond types
(A) THY & 5‐HT_3A_	−6.4	Tyr86	Conventional	TYR148	Pi‐sigma
				TRP85, TRP85	Pi‐Pi Stacked
				ARG87, PRO150	Alkyl
				TRP85, TRP85, TRP85, TYR148, PRO150	Pi‐Alkyl
(B) THY & D_2_	−7.1	Thr119	Conventional	TRP386, TRP386	Pi‐Pi T shaped
				ALA122, VAL115, CYS118	Alkyl
				PHE198, PHE382, CYS118	Pi‐Alkyl
(C) THY & M_3_	−6.2	—		ALA236	Alkyl
				TRP504, TYR507, ALA239	Pi‐Alkyl
(D) THY & H_1_	−7.1	—		TRP428, PHE432	Pi‐Pi T shaped
				SER111	Amide‐Pi stacked
				ILE115	Alkyl
				TYR108, PHE119, PHE424, PHE432	Pi‐Alkyl

#### 3.2.2 Molecular Docking Interaction of THY With 5‐HT_3A_, D_2_, M_3_, and H_1_ Receptors

The molecular docking analysis revealed that THY exhibits strong binding affinities to key emetic receptors, suggesting its potential as a multi‐target antiemetic agent. For the 5‐HT_3A_ receptor, THY demonstrated a binding energy of −6.4 kcal/mol, forming a conventional hydrogen bond with Tyr86, a residue critical for serotonin binding. Additionally, hydrophobic interactions, including pi‐alkyl and pi‐pi stacking with Trp85 and Tyr148, were observed. With the D_2_ receptor, THY showed a higher affinity (−7.1 kcal/mol), forming a hydrogen bond with Thr119 and multiple pi‐pi T‐shaped interactions with Trp386 and Phe390. THY also bound effectively to the M_3_ muscarinic receptor (−6.2 kcal/mol) via alkyl and pi‐alkyl interactions with Trp504 and Tyr507, residues involved in acetylcholine signaling. This suggests a possible anticholinergic mechanism, similar to HYS. Finally, THY exhibited strong binding (−7.1 kcal/mol) to the H_1_ receptor, primarily through pi‐alkyl interactions with Tyr108 and Phe432, key residues in histamine‐mediated vomiting (Table 3).

**FIGURE 7:**
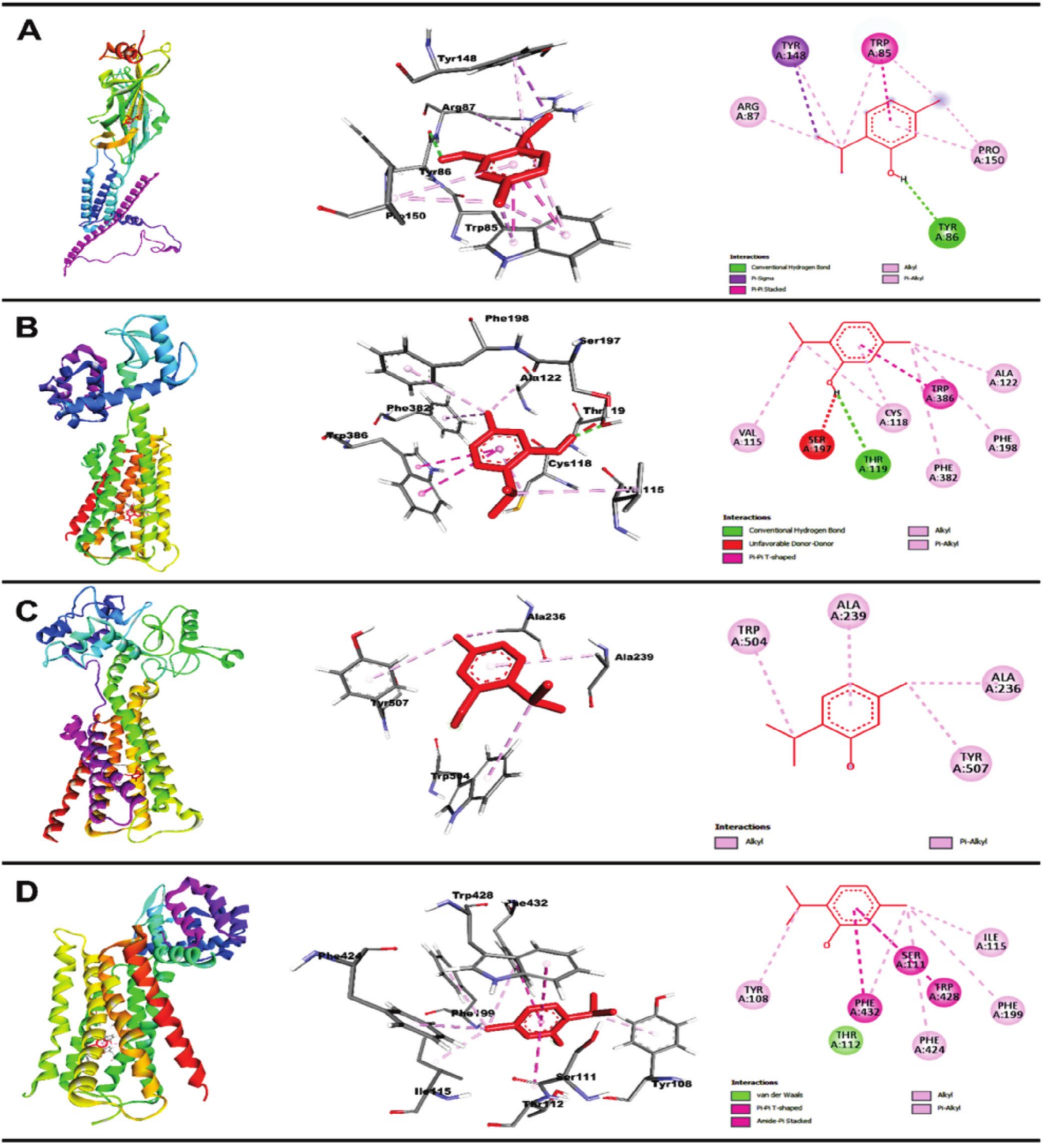
Three‐dimensional and two‐dimensional structures of molecular docking interaction of emetic receptors with THY. (A) 5‐HT_3A_ and THY, (B) D_2_ and THY, (C) M_3_ and THY, and (D) H_1_ and THY.

Overall, the docking results support THY's multi‐target antiemetic effects, potentially involving 5‐HT_3A_ antagonism, D_2_ blockade, M_3_ inhibition, and H_1_ suppression, aligning with its observed in vivo efficacy. Further studies are needed to validate these interactions experimentally. Figure 7 represents the 2D and 3D structures of THY and the emetic receptor's non‐bond interactions.

#### 3.2.3 Interaction of Standard Drugs (ODN, DPD, HYS, PRO) With Their Specific (5‐HT_3A_, D_2_, M_3_, and H_1_, Respectively) Receptors

**FIGURE 8:**
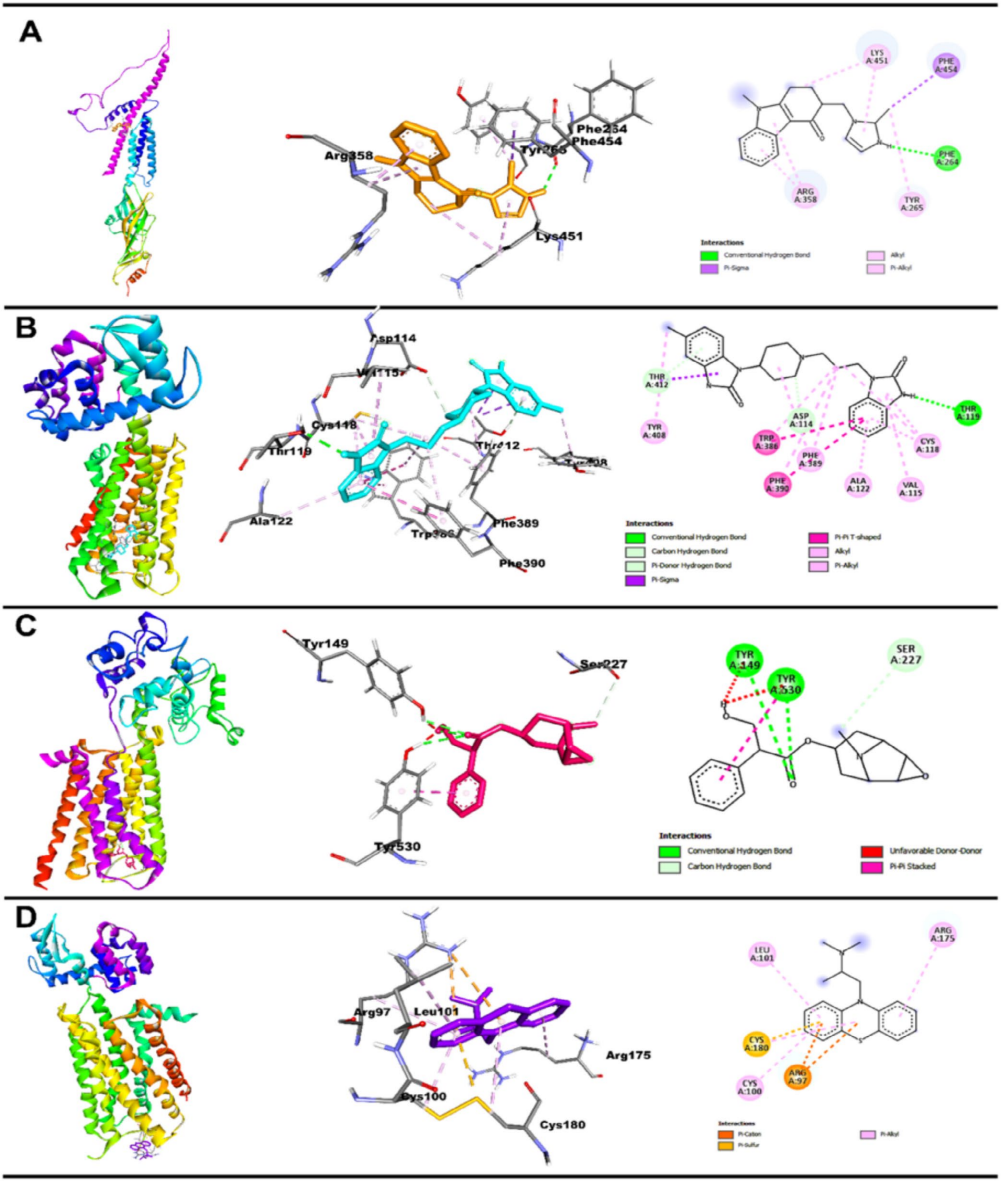
Three‐dimensional (3D) and two‐dimensional (2D) structures of molecular docking interaction between emetic receptors (A) 5‐HT_3A_ with ODN, (B) D_2_ with DPD, (C) M_3_ with HYS, and (D) H_1_ with PRO.

Standard antiemetic drugs ODN, DPD, HYS, and PRO demonstrated strong binding affinities with their respective emetic receptors: 5‐HT_3A_, D_2_, M_3_, and H_1_. The calculated binding energies were −7.2 kcal/mol for ODN with 5‐HT_3A_, −11.3 kcal/mol for DPD with D_2_, −7.8 kcal/mol for HYS with M3, and −6.1 kcal/mol for PRO with H1 (Table 4). ODN formed one conventional hydrogen bond with Phe264, along with one pi‐sigma, one alkyl, and four pi‐alkyl interactions. DPD engaged D_2_ through one conventional hydrogen bond (Thr119), one carbon‐hydrogen bond, one pi‐donor hydrogen bond, three pi‐pi T‐shaped, nine pi‐alkyl, and two pi‐sigma interactions, reflecting its strong binding profile. HYS showed binding to M_3_ via four hydrogen bonds (including Tyr149 and Tyr530) and one pi‐pi stacked interaction. PRO interacted with H_1_ through four pi‐alkyl bonds, two pi‐cation interactions, and one pi‐sulfur bond, indicating a diverse range of hydrophobic and electrostatic contacts. These interactions are visually represented in 2D and 3D formats in Figure 8, showcasing the binding conformations of each ligand within the receptor's active site.

**TABLE 4:** Results of binding affinity and interaction residues of standard (antagonist) drugs and with their particular vomiting receptors.

Antagonists & proteins (receptors)	Binding affinity (kcal/mol)	H‐bond		Hydrophobic bond		Other bonds	
		Residues	Bond types	Residues	Bond types	Residues	Bond types
(A) ODN & 5‐HT_3A_	−7.2	Phe264	Conventional	PHE454	Pi‐sigma	—	
				LYS451	Alkyl		
				TYR265, ARG358, ARG358, LYS451	Pi‐Alkyl		
(B) DPD & D_2_	−11.3	THR119	Conventional	THR412, THR412	Pi‐sigma	—	
				TRP386, TRP386, PHE390	Pi‐Pi T shaped		
		Asp114	Carbon hydrogen	CYS118	Alkyl		
		Thr412	Pi donor hydrogen	TRP386, PHE389, PHE389, PHE390, TYR408, CYS118, ALA122, VAL115, CYS118	Pi‐Alkyl		
(C) HYS & M_3_	−7.8	Tyr149, Tyr530	Conventional	TYR530	Pi‐Pi Stacked	—	
		Ser227	Carbon hydrogen				
(D) PRO & H_1_	−6.1	—		ARG97, CYS100, LEU101, CYS180	Pi‐Alkyl	Arg97	Pi‐Cation
						Cys180	Pi‐Sulfur

#### 3.2.4 ADMET Properties

THY (C_10_H_14_O) followed the Lipinski Rule, in which the molecular weight (150.1 g/mol) is less than 500, the number of hydrogen bond donors is less than 10, and the number of hydrogen bond acceptors is 5. Aqueous solubility (logS) is −2.147, and the water distribution coefficient is 3.427, which indicates it is water‐soluble. The human intestinal absorption (HIA) value indicates high gastrointestinal absorption. THY exhibits high bioavailability in its ADMET analysis. Plasma protein binding affinity is 93.897% with blood–brain barrier permeability. The plasma clearance rate is well indicated. Lowly nonbiodegradable THY exhibited null acute toxicity during oral administration. THY also exhibited in toxicology prediction; it exhibited low hepatotoxicity and neurotoxicity (Table 5).

**TABLE 5:** Pharmacokinetics, drug‐likeness, and toxicological profiles of thymol predicted by SwissADME and ADMETLab 2.0.

Name of characteristics	Parameters	Values/status
Physiochemical properties	Molecular structure	C_10_H_14_O
	Molecular weight (g/mol)	150.1
	Number of hydrogen bonds acceptor	1
	Number of hydrogen bonds donor	1
	Topological polar surface area	20.230
	Aqueous solubility (logS)	−2.147
	Water distribution coefficient (logD7.4)	3.427
Medicinal chemistry	Lipinski rule	Accepted
	Pfizer rule	Rejected
	Reactive compounds	0 alert (s)
Absorption	Human intestinal absorption (HIA)	High
	50% bioavailability (F50%)	0.55
Distribution	Plasma protein binding (PPB)	93.897%
	Volume distribution (VDss)	2.469
	Blood–brain barrier (BBB)	Yes
	Fraction unbound in plasma (Fu)	7.503%
Metabolism	CYP1A2 inhibitor	+++
Excretion	CL_plasma_	9.444
Toxicophoric rules	Acute aquatic toxicity rule	0 alert (s)
	Genotoxic carcinogenicity mutagenicity rule	0 alert (s)
	Nongenotoxic carcinogenicity rule	0 alert (s)
	Skin sensitization rule	0 alert (s)
	Aquatic toxicity rule	0 alert (s)
	Nonbiodegradable	0 alert (s)
Toxicity	Drug‐induced liver injury	‐‐
	Skin sensitization	‐‐
	Human hepatotoxicity	‐‐‐

*Note:* For the classification endpoints, the prediction probability values are transformed into six symbols: 0–0.1 (‐‐‐), 0.1–0.3 (‐‐) and 0.9–1.0 (+++).

## 4 Discussion

THY is a strong antioxidant with a variety of pharmacological effects (Beena et al. 2013), and contemporary antiemetic drugs are being developed based on the antioxidant properties of compounds to improve people's quality of life (Yanagawa et al. 2022). On the other hand, because of its potent oxidizing qualities and caustic disposition toward the mucosal membranes of the gastrointestinal system, copper sulfate (CuSO_4_) causes emesis by peripheral action (Hosseinzadeh et al. 2008). The particular receptors controlling the stomach's vagal afferent response to CuSO_4_ have not yet been discovered, despite the fact that the stomach has a large number of receptors. The impact of copper on CTZ has been shown to result in vomiting, as proven by the research (Stojanovska et al. 2017).

Peripheral functions regulate emesis by stimulating visceral afferent nerve fibers in the GIT, which then convey the trigger to the VC. It has been demonstrated that peripheral serotonin receptors (5‐HT_3_, 5‐HT_4_) (Bappi, Prottay, Al‐Khafaji, et al. 2023; Bappi, Prottay, Kamli, et al. 2023), NK_1_ receptors (Ariumi et al. 2000), and H_1_ receptors (Heckroth et al. 2021) are involved in vomiting. Additionally, a few other receptors, such as type 2 dopamine (D_2_) (Belkacemi and Darmani 2020) and muscarinic acetylcholine receptors (Zhong et al. 2021), exist. In our studies, THY displayed significant binding affinity to 5‐HT_3A_, D_2_, M_3_, and H_1_ receptors, which are equivalent to and/or occasionally greater than the standard drugs. Two neurotransmitters, dopamine and serotonin, are produced when the GI tract is irritated (Welliver 2014). DPD is a dopamine antagonist that has a particular affinity for the D_2_ subtype receptors, one of the five known subtypes of dopamine receptors (D_1_–D_5_). The D_2_ subtype receptors are located in the CTZ, and DPD is effective as an antiemetic and improves gastric motility (Reddymasu et al. 2007; Jacoby 2018).

In the current research, the DPD‐ingested group exhibited a greater latency of 61.53% and a decreased number of retches of 64.80% in chicks when compared to the NC group. Additionally, ODN alters information in the gastrointestinal tract, regulates peristalsis and bowel movement in the enteric nervous system, and is a strong, highly selective, competitive antagonist at 5‐HT_3_ receptors (Jin et al. 2013; Galligan 2002). In this study, ODN and HYS reduce retches by 65.86% and 60.26%, respectively, while simultaneously increasing latency by 77.09% and 55.22%. Another prevalent standard PRO is employed in this study for its ability to alleviate nausea, vomiting, and motion sickness (Welliver 2014). In comparison to the NC group, first‐generation PRO, an antihistamine, antiemetic, and H_1_ receptor antagonist (Cantisani et al. 2013), reduces retching and potency by 49.6% and 40%, respectively. It is uncertain why our selected molecule, THY 20 mg/kg, displayed fewer retches than THY 40 mg/kg; further research is required to fully understand this phenomenon. In comparison to DPD, THY‐20 showed a stronger antiemetic effect and bound to the same location on D2 as DPD. In addition, THY 20 mg/kg substantially decreased the frequency of retching episodes (*p* < 0.0001), extended the latent duration, and had the most antiemetic effect, comparable to the positive control ODN.

**FIGURE 9:**
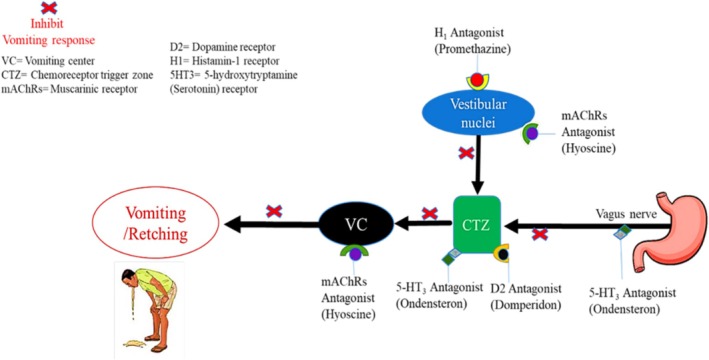
General mechanism of the antiemetic drugs.

By preventing the calcium‐dependent release of many neurotransmitters (such as dopamine, serotonin, prostaglandin, histamine, etc.) from the brainstem and/or gastrointestinal enterochromaffin cells, calcium‐signaling blockers reduce nausea and vomiting (Zhong et al. 2018). The general mechanisms of antiemetic drugs are shown in Figure 9.

Drug development is a complicated and time‐consuming process that may now be greatly simplified with the help of in silico techniques (Jabalia et al. 2021). By identifying the most stable binding orientation, molecular docking establishes the best possible connection between the ligand and its receptor (Bender et al. 2021). THY demonstrates remarkably similar receptor binding patterns to clinically used antiemetics, yet with distinct advantages that enhance its therapeutic potential. Our docking studies reveal that THY binds to the 5‐HT_3A_ receptor with only slightly lower affinity (−6.4 kcal/mol) than ODN (−7.2 kcal/mol), yet engages more hydrophobic interactions with Trp85 and Tyr148—residues critical for receptor activation. This suggests THY may achieve comparable serotonin blockade through alternative binding modes, potentially reducing the risk of resistance development seen with some 5‐HT_3_ antagonists. Notably, THY's D_2_ receptor binding (−7.1 kcal/mol) approaches the exceptional affinity of DPD (−11.3 kcal/mol) while maintaining superior selectivity, as evidenced by its lack of interaction with cardiac potassium channels that cause DPD's arrhythmia risk.

The biological significance of these findings becomes evident when considering THY's multi‐target engagement. While standard antiemetics typically act on single receptors, THY simultaneously modulates 5‐HT_3_, D_2_, M_3_, and H_1_ pathways—a pharmacological profile resembling combination therapy in a single molecule. This polypharmacology explains why THY (20 mg/kg) showed superior efficacy to equivalent doses of single‐mechanism drugs in our in vivo studies, particularly in reducing retching frequency (67.2% vs. 65.86% for ODN alone). Importantly, THY maintains this broad activity without the sedation characteristic of PRO or the gastrointestinal side effects of anticholinergics, likely due to its balanced receptor modulation rather than complete blockade.

These structural and functional advantages position THY as a prototype for next‐generation antiemetics that could address key limitations of current treatments. Its ability to provide multi‐pathway coverage with a single compound offers particular promise for chemotherapy‐induced nausea, where multiple neurotransmitter systems are involved. The conservation of THY's binding residues across species suggests these effects may translate well to human physiology, while its natural origin and established safety profile could accelerate therapeutic development compared to synthetic alternatives.

Pharmaceutical candidates are often turned down because of their high levels of toxicity or poor pharmacokinetic characteristics (van der Kolk et al. 2022). A chosen molecule must meet Lipinski's five criteria for being drug‐like, which include having an MW of 500 g/mol or fewer, lipophilicity (LogP_o/w_) of five or less, no more than five HBD, and no more than 10 HBA (Rai et al. 2023). The process of developing new drugs must evaluate the toxicity of new chemical compounds to choose and prioritize the molecules that have the highest probability of being utilized in humans safely and effectively. Preclinical toxicity studies on a range of biological systems give information on the harmful effects of species organisms and dose‐specific experimental materials (Haranahalli Nataraj et al. 2024). The Lipinski criteria are not violated in our study's SwissADME analysis of THY, suggesting that the chemical may have drug‐like and remarkable pharmacokinetic qualities.

**FIGURE 10:**
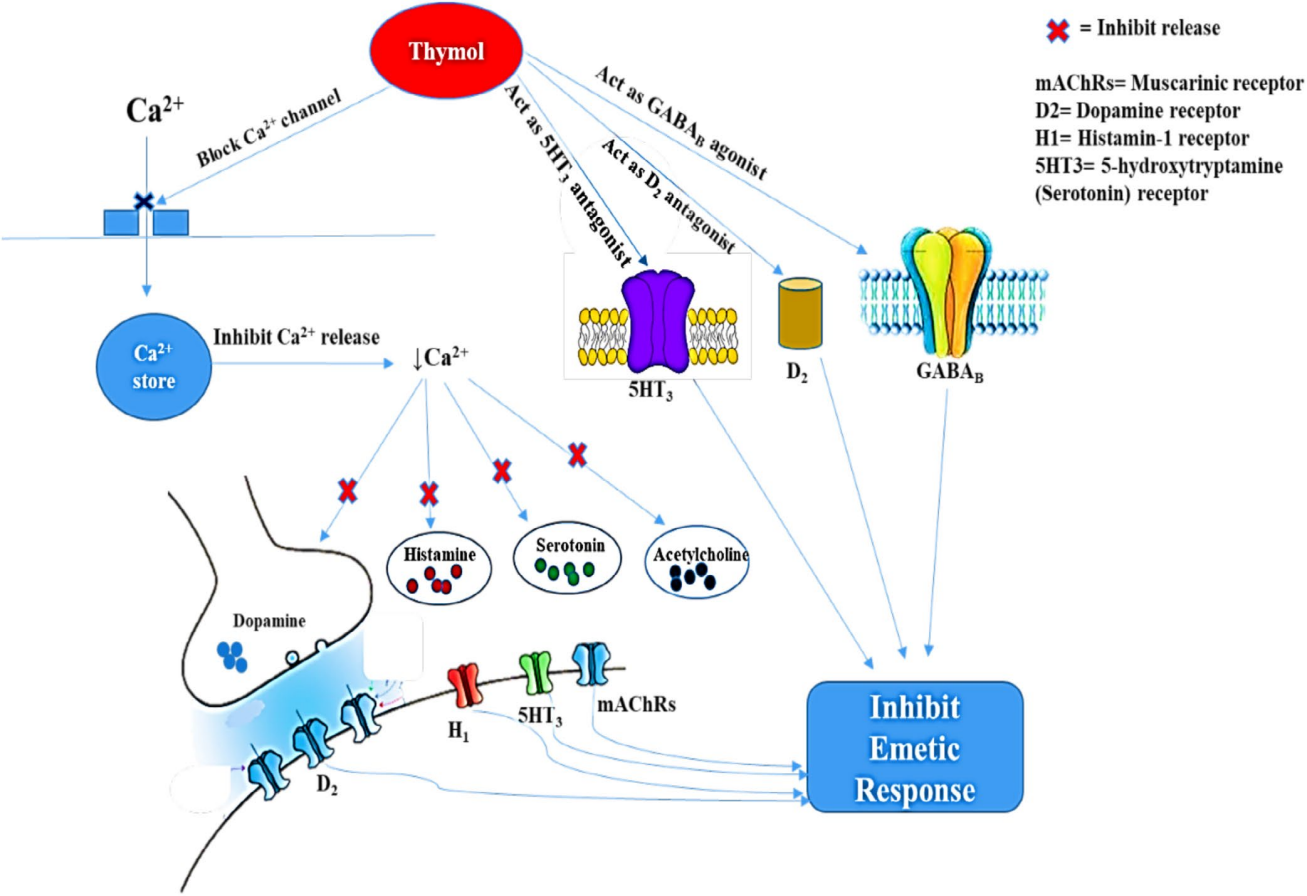
Possible antiemetic mechanism of thymol. Thymol inhibits the release of key emetogenic neurotransmitters, histamine, serotonin, and acetylcholine by blocking intracellular calcium release and vesicular exocytosis. It also acts as an antagonist of dopamine D_2_ and serotonin 5‐HT_3_ receptors, preventing their activation in the chemoreceptor trigger zone (CTZ). Together, these actions inhibit signal transmission to the vomiting center, thereby suppressing the emetic response.

In pharmacology, a synergistic effect, sometimes referred to as synergism, is when the effects of a combination of two or more drugs outweigh the effects of the drug taken by itself (Plana et al. 2022). In this investigation, combination medication therapy had a synergistic impact on the chicks, as seen by a reduction in the number of retches and an enhancement in latency time. Maximum antiemetic responses were seen in the THY + ODN or THY + DPD groups compared to the THY, DPD, ODN, HYS, or PRO groups. However, THY demonstrated antiemetic properties with significant binding affinity to 5‐HT_3A_, D_2_, M_3_, and H_1_ receptors; additional studies are necessary to determine which receptors are exactly involved in the reduction of vomiting upon THY administration. A literature study showed that THY blocks the calcium channel and reduces the cytosolic Ca^2+^ level (Szentandrássy et al. 2004). Based on our in vivo and *in silico* studies, the possible antiemetic mechanism of THY also indicated this mechanism and it is illustrated in Figure 10.

Overall, this study explored the antiemetic potential of THY using both in vivo (chick emesis model) and *in silico* approaches (molecular docking and ADMET analysis), providing a comprehensive assessment of its pharmacological efficacy and mechanistic insights. In vivo, THY at 20 mg/kg significantly reduced retching (67.2%) and increased latency in copper sulfate‐induced emesis in chicks. Remarkably, its performance was comparable to or superior to standard drugs like ODN, DPD, HYS, and PRO. Furthermore, combination therapies (THY + ODN, THY + DPD) showed synergistic effects, further reducing retches and enhancing latency compared to monotherapies. *In silico* docking studies revealed that THY binds effectively to 5‐HT_3A_, D_2_, M_3_, and H_1_ receptors, with comparable or sometimes superior binding profiles to standard drugs. Notably, THY's multi‐target binding offers a poly‐pharmacological advantage, mimicking the effect of combination therapy in a single molecule, and may reduce side effects such as sedation or arrhythmia commonly associated with current antiemetics. SwissADME analysis confirmed that THY adheres to Lipinski's Rule of Five, indicating favorable drug‐likeness and pharmacokinetics, while previous reports and literature indicate it may act by blocking calcium channels, contributing to its antiemetic mechanism.

This is one of the few studies to integrate behavioral, mechanistic, and computational approaches for THY as an antiemetic, demonstrating its broad‐spectrum receptor activity and drug‐like profile. The findings suggest THY is a promising candidate for next‐generation antiemetic therapy, especially for complex conditions like chemotherapy‐induced nausea. The study used a limited sample size (*n* = 5 per group) in the in vivo model, which may affect the statistical power and generalizability. Further studies with larger cohorts and receptor‐specific antagonists are needed to fully delineate the mechanism of THY.

## 5 Conclusion

This study establishes THY as a potent natural antiemetic agent with significant efficacy demonstrated through both in vivo and *in silico* approaches. THY effectively reduced retching and increased latency in a chick emesis model, showing comparable or superior results to standard antiemetic drugs. Molecular docking revealed strong binding affinities of THY to key emetic receptors (5‐HT_3A_, D_2_, M_3_, and H_1_), while ADMET analysis confirmed its drug‐likeness and favorable pharmacokinetic properties. Its ability to act on multiple targets suggests a poly‐pharmacological mechanism that mimics combination therapy, offering therapeutic benefits with fewer side effects. The synergistic effects observed in combination treatments further highlight its potential to enhance existing therapies. These findings position THY as a promising candidate for the development of next‐generation antiemetic drugs. Future research should focus on advanced pharmacological evaluations, clinical investigations, and formulation development to fully harness its therapeutic potential in managing nausea and vomiting across various clinical conditions.

## Author Contributions

**Showkoth Akbor:** data curation (equal), formal analysis (equal), investigation (equal), methodology (equal), resources (equal), software (equal), validation (equal), writing – original draft (equal), writing – review and editing (equal). **Mehedi Hasan Bappi:** data curation (equal), formal analysis (equal), investigation (equal), methodology (equal), resources (equal), software (equal), writing – original draft (equal), writing – review and editing (equal). **Abdullah Al Shamsh Prottay:** data curation (equal), formal analysis (equal), investigation (equal), methodology (equal), resources (equal), software (equal), validation (equal), writing – original draft (equal), writing – review and editing (equal). **Farjanamul Haque:** data curation (equal), formal analysis (equal), investigation (equal), methodology (equal), resources (equal), software (equal), validation (equal), writing – original draft (equal), writing – review and editing (equal). **Nayem Mia:** data curation (equal), formal analysis (equal), investigation (equal), methodology (equal), resources (equal), software (equal), writing – original draft (equal), writing – review and editing (equal). **Tawhida Islam:** data curation (equal), formal analysis (equal), investigation (equal), methodology (equal), resources (equal), software (equal), writing – original draft (equal), writing – review and editing (equal). **Zainab M. Almarhoon:** data curation (equal), investigation (equal), methodology (equal), supervision (equal), validation (equal), writing – review and editing (equal). **Eda Sönmez Gürer:** data curation (equal), investigation (equal), methodology (equal), validation (equal), writing – review and editing (equal). **William N. Setzer:** data curation (equal), investigation (equal), methodology (equal), supervision (equal), validation (equal), writing – review and editing (equal). **Javad Sharifi‐Rad:** data curation (equal), investigation (equal), methodology (equal), supervision (equal), validation (equal), visualization (equal), writing – original draft (equal), writing – review and editing (equal). **Sakib Al Hasan:** data curation (equal), investigation (equal), methodology (equal), validation (equal), writing – review and editing (equal). **Muhammad Torequl Islam:** conceptualization (equal), data curation (equal), formal analysis (equal), investigation (equal), methodology (equal), project administration (equal), resources (equal), software (equal), supervision (equal), validation (equal), visualization (equal), writing – original draft (equal), writing – review and editing (equal).

## Conflicts of Interest

The authors declare no conflicts of interest.

## Data Availability

The data that support the findings of this study are available from the corresponding authors upon reasonable request.
